# Lung-specific sulfonium lipid nanoparticle formulation of dexamethasone suppresses endotoxin-induced lung inflammation

**DOI:** 10.3389/fimmu.2026.1821659

**Published:** 2026-05-29

**Authors:** Yuqin Men, Chunyan Wang, David O. Popoola, Zhi Cao, Weikun Tian, Robert N. Cooney, Qinghe Meng, Yamin Li

**Affiliations:** 1 Department of Pharmacology, State University of New York, Upstate Medical University, Syracuse, NY, United States; 2 Department of Surgery, State University of New York, Upstate Medical University, Syracuse, NY, United States; 3 Sepsis Interdisciplinary Research Center (SIRC), State University of New York, Upstate Medical University, Syracuse, NY, United States

**Keywords:** acute lung injury, dexamethasone, ARDS, lipid nanoparticle, lung-targeting

## Abstract

**Introduction:**

Acute lung injury (ALI) and acute respiratory distress syndrome (ARDS) represent a spectrum of acute respiratory failure arising from the same underlying pathophysiological processes and are associated with substantial morbidity and mortality worldwide. Although corticosteroids such as Dexamethasone are commonly administered to patients with moderate-to-severe ARDS, their clinical benefit remains controversial, and systemic administration is often associated with significant adverse effects.

**Methods:**

We developed a targeting ligand-free, sulfonium lipid nanoparticle (sLNP)-based drug delivery system, Dex/DOSEH, for intravenous lung-targeted delivery of dexamethasone.

**Results:**

In a lipopolysaccharide (LPS)-induced murine ALI model, treatment with Dex/DOSEH formulation significantly reduced proinflammatory cytokine production, decreased immune cell infiltration, preserved capillary-alveolar barrier integrity, and attenuated histopathological lung injury compared with controls.

**Discussion:**

Our findings demonstrate that Dex/DOSEH drug formulation enables effective lung-targeted delivery of dexamethasone and achieves robust anti-inflammatory therapeutic efficacy in ALI. This platform represents a promising therapeutic strategy for the treatment of ALI/ARDS.

## Introduction

Acute lung injury (ALI) comprises a group of severe inflammatory conditions of the lung triggered by diverse insults (1, 2). Acute respiratory distress syndrome (ARDS) represents the most severe form of ALI and predominantly affects critically ill patients (3, 4). Despite substantial advances in supportive care and pharmacological management, ALI/ARDS remains a leading cause of morbidity and mortality globally (4–6). Corticosteroids, including dexamethasone (Dex), have been used to mitigate pulmonary inflammation; however, their efficacy in clinical settings remains controversial, largely due to adverse effects associated with systemic administration. These limitations highlight the need for more effective and targeted therapeutic strategies (7–11).

In the DEXA-ARDS trial (ClinicalTrials.gov, NCT01731795), early intravenous (i.v.) administration of Dex significantly reduced the duration of mechanical ventilation and overall mortality in patients with moderate-to-severe ARDS (12). The CoDEX trial (ClinicalTrials.gov, NCT04327401) demonstrated that i.v. Dex, in addition to standard care, increased ventilator-free days in patients with COVID-19-associated moderate-to-severe ARDS, although it did not significantly reduce all-cause mortality or other prespecified secondary outcomes (13). Beyond variability in efficacy, systemic corticosteroid therapy is frequently associated with various adverse effects, such as hyperglycemia, increased susceptibility to infection, gastrointestinal complications, myopathy, psychiatric disturbances, osteoporosis and fractures, and adrenal suppression (8, 9). These observations suggest that suboptimal drug biodistribution following systemic administration may contribute to both insufficient therapeutic benefit and systemic adverse effects. Consequently, local or organ-targeted delivery strategies that preferentially accumulate the drug at sites of interest may offer a promising approach to enhance efficacy while minimizing off-target effects.

Nanocarrier-based targeted delivery of Dex has been explored in rheumatoid arthritis (14–16), bone regeneration (17), neurological disorders (18), gastrointestinal diseases (19), pulmonary inflammation (20), and other conditions (21–24). For example, Li et al. developed anti-intercellular adhesion molecule-1 (anti-ICAM-1)-conjugated nanostructured lipid carriers (NLCs) to target the pulmonary endothelium and deliver Dex (25). Antibody functionalization markedly enhanced pulmonary accumulation of the NLCs, and in a lipopolysaccharide (LPS)-induced ALI mouse model, this system reduced proinflammatory cytokine expression and alleviated tissue injury (25). Similarly, Chen et al. reported a solid lipid nanoparticle (SLN) formulation incorporating Dex-palmitate; following nebulized administration in an LPS-induced lung injury model, this formulation reduced tumor necrosis factor-α (TNF-α) and interleukin-6 (IL-6) levels in lung tissue and improved histopathological outcomes (26). While these studies underscore the promise of targeted Dex delivery in ALI, strategies that rely on targeting ligands or local administration routes may pose challenges for clinical translation.

**Figure 1:**
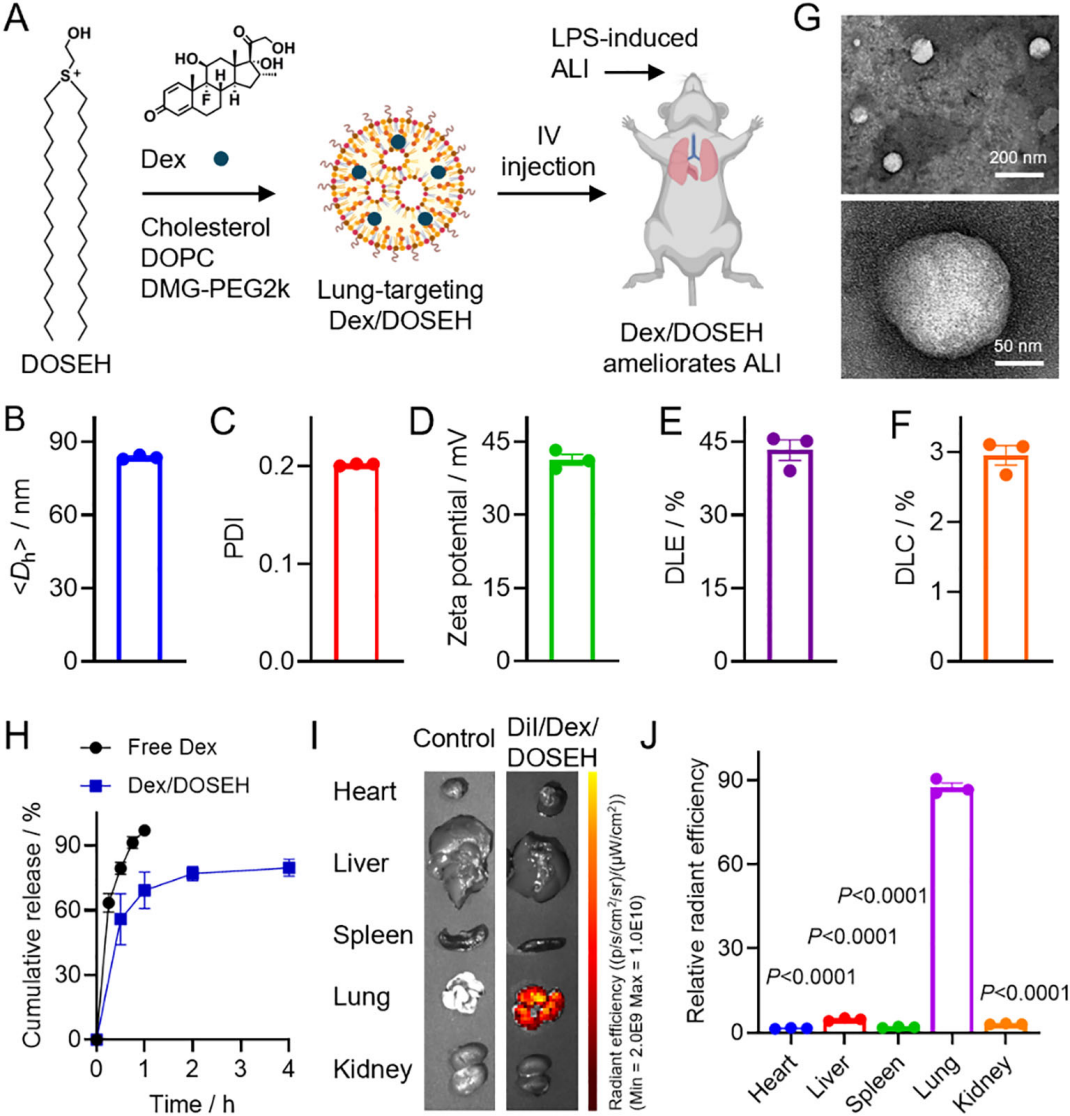
Physicochemical characterization and biodistribution of Dex/DOSEH formulation. **(A)** Schematic illustration of DOSEH sLNP-mediated Dex delivery for the treatment of LPS-induced lung inflammation. Physicochemical property characterization of the Dex/DOSEH formulation, including **(B)** <*D*_h_>, **(C)** PDI, **(D)** zeta potential, **(E)** DLE, and **(F)** DLC. N = 3, mean ± SEM. **(G)** Representative negatively stained TEM images. **(H)** *In vitro* drug release profile of Dex/DOSEH sLNPs. **(I)** Ex vivo IVIS epifluorescence imaging of major organs and **(J)** quantification of relative radiant efficiency across different organs. N = 3, mean ± SEM, one-way ANOVA, *P* values indicate comparisons relative to lung tissue.

In this study, we aimed to develop a targeting ligand-free lipid nanoparticle (LNP) formulation for lung-selective delivery of Dex via i.v. administration as a potential therapeutic strategy for ALI/ARDS (**Figure 1A**). Dex was encapsulated into a DOSEH-based sulfonium lipid nanoparticle (sLNP; Dex/DOSEH), which demonstrated selective accumulation in lung tissue following systemic delivery. After physicochemical characterization of Dex/DOSEH, we evaluated its therapeutic potential in a mouse model of endotoxin-induced lung inflammation and injury. Our results demonstrate that lung-targeted delivery of Dex using the ligand-free DOSEH sLNP system effectively attenuates pulmonary inflammation and tissue damage, supporting its potential as a promising treatment modality for ALI/ARDS.

## Materials and methods

### General

All chemicals and solvents used for lipid synthesis were obtained from Sigma-Aldrich, Thermo Fisher Scientific, and Oakwood Chemical. 1,2-dioleoyl-sn-glycero-3-phosphocholine (DOPC) and 1,2-dimyristoyl-rac-glycerol-methoxypolyethylene glycol-2000 (DMG-PEG2k) were purchased from Avanti Polar Lipids. Cholesterol and LPS were purchased from Sigma-Aldrich, and Dex was obtained from TCI Chemicals. The fluorescent dye 1,1′-dioctadecyl-3,3,3′,3′-tetramethylindocarbocyanine perchlorate (DiI) was purchased from Thermo Fisher Scientific. The sulfonium lipid DOSEH was synthesized according to previously reported procedures (27, 28).

### Preparation of Dex/DOSEH formulations

Dex-loaded DOSEH sulfonium lipid nanoparticles (Dex/DOSEH) were prepared using a self-assembly method. Briefly, DOSEH, cholesterol, DOPC, DMG-PEG2k, and Dex were dissolved in ethanol at a weight ratio of 40:9.6:4.8:2.4:4 (DOSEH:cholesterol:DOPC: DMG-PEG2k:Dex). The weight percentage of feeding Dex relative to total lipids (DOSEH, cholesterol, DOPC, and DMG-PEG2k) is approximately 7% (4/(40 + 9.6 + 4.8 + 2.4) × 100%). The ethanol solution was rapidly mixed with 25 mM NaOAc buffer (Sigma-Aldrich) under vigorous vortexing to induce nanoparticle self-assembly. The resulting suspension was then dialyzed against distilled water using dialysis tubing (MWCO 12–14 kDa; Spectra Por) to remove residual ethanol. For fluorescent labeling, DiI-loaded Dex/DOSEH (DiI-Dex/DOSEH) were prepared using the same procedure, with DiI added to the initial ethanol phase at 2.5 wt% relative to total lipid content (DOSEH, cholesterol, DOPC, and DMG-PEG2k). All formulations were freshly prepared and used immediately for subsequent experiments.

### Physicochemical characterization

The average hydrodynamic diameter (<*D*_h_>), polydispersity index (PDI), and zeta-potential of Dex/DOSEH were measured at room temperature using a Malvern Zetasizer Ultra. Nanoparticle morphology was examined by transmission electron microscopy (TEM) using a JEOL JEM-1400 microscope following negative staining with 1% (w/v) phosphotungstic acid (pH 7.0).

Drug loading efficiency (DLE%; (mass of encapsulated drug)/(mass of feeding drug) × 100%) and drug loading content (DLC%; (mass of encapsulated drug)/(mass of carrier + mass of encapsulated drug) × 100%) were quantified following previously established methods (29). A standard calibration curve for Dex was generated using high-performance liquid chromatography (HPLC). HPLC analysis was performed on a Waters 1525 binary pump system equipped with a 2489 UV-visible detector and an XBridge™ column. The mobile phase consisted of water and acetonitrile (1:9, v/v) at a flow rate of 1.0 mL/min. *In vitro* drug release was performed using a dialysis method in phosphate-buffered saline (PBS, pH 7.4) at 37 °C with continuous stirring, following our previously reported procedures (29).

### Animal studies

Male and female C57BL/6 mice (8–10 weeks of age) were purchased from The Jackson Laboratory. Animals were inbred and housed in the SUNY Upstate Department of Laboratory Animal Resources (DLAR) facility under controlled conditions (22 °C; 12-h light/12-h dark cycle) with free access to food and water. All animal experiments were approved by the Institutional Animal Care and Use Committee (IACUC No. 513) of SUNY Upstate Medical University and conducted in accordance with NIH guidelines and ARRIVE recommendations.

### *In vivo* biodistribution imaging

For biodistribution analysis, mice received DiI-Dex/DOSEH via intravenous retro-orbital injection at a dose of 6.5 mg/kg. 30 minutes post-administration, mice were euthanized and major organs were surgically excised for ex vivo epifluorescence imaging. Images were acquired using an IVIS 50 imaging system (PerkinElmer) at SUNY Upstate Medical University. Fluorescence signals were analyzed using the Living Image^®^ software.

### Cytotoxicity measurement

For HPAEC and A549 cell viability analysis, 5,000 cells per well with 100 µL of cell culture medium (Gibco) supplemented with 10% FBS (VWR) and 1% PS (Gibco) were used. 24 h after cell seeding, Dex/DOSEH or corresponding amount of free Dex or empty DOSEH sLNP were added to the cell culture media with a final Dex/DOSEH concentration of 3, 10, or 30 µg/mL. The cells were then incubated at 37 °C with 5% CO_2_ for another 24 hours. The MTS Assay kit (Abcam) was used to measure cell viability according to the manufacturer’s guidelines.

### Safety evaluation

To assess systemic toxicity, mice were euthanized 7 days following intravenous administration of Dex/DOSEH (0.1 mg Dex/kg body weight), and blood samples were collected. Plasma levels of alanine aminotransferase (ALT), aspartate aminotransferase (AST), blood urea nitrogen (BUN), and creatinine were measured using commercially available assay kits (MilliporeSigma) according to the manufacturers’ protocols.

### Induction of acute lung injury and treatment

Acute lung injury was induced using an established LPS model. Briefly, mice were anesthetized with ketamine and xylazine and placed in a supine position. LPS (2.5 mg/kg) or vehicle control was administered via intratracheal instillation using PE-10 tubing connected to an insulin syringe. Intravenous administration of Dex/DOSEH was performed two hours prior to LPS stimulation. Following anesthesia induction with 2.5% isoflurane, a sterile insulin syringe was inserted into the medial canthus of the eye at a 30-45° angle, and the free Dex (0.4 mg/kg) or Dex/DOSEH formulation (0.1 mg/kg Dex and ca. 3.3 mg/kg DOSEH sLNP carrier) was slowly injected into the retro-orbital venous sinus.

### Sample collection and analysis

Mice were euthanized 24 h after LPS challenge and blood was collected. Bronchoalveolar lavage fluid (BALF) was obtained by lavaging the lungs with three sequential aliquots of 0.5 mL sterile saline. Concentrations of TNF-α and IL-6 in serum and BALF were quantified using ELISA kits (Invitrogen) according to the manufacturer’s instructions.

For cytological analysis, BALF samples were centrifuged at 250 × g for 10 min to pellet cells. Cell pellets were resuspended in 1 mL sterile saline, and 100 µL of the suspension was cytospun onto glass slides at 1000 rpm for 3 min. Slides were air-dried and stained with Hema-3. Neutrophils and macrophages were counted in 20 high-power fields (HPF) by two blinded observers using a microscope (Nikon Eclipse TE2000-U). Total protein concentration in BALF was measured using a micro-BCA assay kit (Thermo Scientific).

For histological evaluation, lung tissues were fixed, paraffin-embedded, sectioned at 5 µm, and stained with hematoxylin and eosin (H&E). Lung injury was scored using a semi-quantitative system described previously (30), assessing neutrophil infiltration, hyaline membrane formation, proteinaceous debris, and alveolar septal thickening. Each parameter was scored from 0 to 2, and a composite injury score was calculated by summation and normalization to the number of fields analyzed. Three representative regions per section were evaluated across 20 HPFs at ×400 magnification.

### Statistical analysis

Data are presented as mean ± standard deviation (SD) or mean ± standard error of the mean (SEM), as indicated in figure captions. Sample sizes are specified in the corresponding figure legends. Statistical analyses were performed using GraphPad Prism 10 software. Comparisons between two groups were conducted using Student’s t-test, while comparisons among three or more groups were performed using one-way analysis of variance (ANOVA). All analyses were conducted without blinding. A *P* value < 0.05 was considered statistically significant.

## Results and discussion

### Characterization of Dex/DOSEH formulation

Dex/DOSEH sLNPs exhibited an average hydrodynamic diameter (<*D*_h_>) of approximately 83.7 nm, comparable to previously reported DOSEH-based formulations, indicating that incorporation of Dex did not substantially affect nanoparticle self-assembly (**Figure 1B**) (28). The polydispersity index (PDI) was approximately 0.2, consistent with a relatively monodisperse particle population (**Figure 1C**). The zeta-potential was measured at approximately +41.3 mV (**Figure 1D**), reflecting a highly positive surface charge that is characteristic of sulfonium lipid-based nanoparticles (27, 28). This permanent positive charge arises from the quaternary sulfonium head group and is distinct from traditional amine-based ionizable lipids, whose charge state depends on pKa values and local microenvironmental pH (31–33). Emerging evidence suggests that surface charge plays a critical role in determining organ tropism following systemic administration (34). In particular, positively charged LNPs have been associated with preferential lung accumulation, potentially due to the formation of distinct protein coronas that differ from those formed on neutral or negatively charged nanoparticles (35).

Drug loading efficiency (DLE) and drug loading content (DLC) were determined to be approximately 43% and 3.0%, respectively (**Figures 1E, F**). These values indicate that Dex, which shares structural similarities with cholesterol, can be efficiently incorporated into sLNPs through self-assembly, corroborating prior reports involving amine-based ionizable LNP systems (36). Transmission electron microscopy (TEM) further confirmed that Dex/DOSEH nanoparticles possessed a spherical morphology (**Figure 1G**), consistent with previously reported DOSEH drug formulations (29). It should be noted that DLS measures the hydrodynamic particle size, whereas TEM captures particles in a dehydrated state. As a result, discrepancies between DLS- and TEM-derived sizes are expected. In this study, TEM was used for morphological characterization only, and no quantitative size comparison was performed. *In vitro* release studies demonstrated that Dex/DOSEH exhibited a more sustained release profile compared with free Dex, which displayed rapid burst release (**Figure 1H**). Although *in vitro* release assays cannot fully recapitulate the complexity of *in vivo* environments, this sustained release behavior may be advantageous for maintaining therapeutically relevant drug concentrations at sites of lung injury, potentially prolonging anti-inflammatory effects relative to free drug.

Consistent with our prior studies demonstrating lung-targeted delivery of both mRNA and the small-molecule therapeutic sparstolonin B (SsnB) using ligand-free DOSEH sLNPs, we next evaluated the biodistribution of Dex/DOSEH (28, 29, 37). For this purpose, Dex/DOSEH was fluorescently labeled with DiI (DiI/Dex/DOSEH) and administered intravenously to mice. Ex vivo epifluorescence imaging revealed predominant accumulation of fluorescence signals in the lungs (**Figure 1I**). Quantitative analysis showed that lung fluorescence intensity was approximately 87.6-fold higher than that observed in PBS-injected control lungs and significantly greater than that detected in other major organs, including the heart, liver, spleen, and kidneys (**Figure 1J**). These findings corroborate our previous observations and further support the lung-selective accumulation of Dex/DOSEH following systemic administration. In contrast to free Dex, which lacks inherent tissue selectivity and is expected to distribute broadly throughout the body, Dex/DOSEH enables preferential delivery to the pulmonary microenvironment. Such targeted accumulation may enhance local drug concentration at sites of inflammation while limiting off-target exposure, thereby improving the therapeutic index.

### Cytotoxicity and safety evaluation of Dex/DOSEH

**Figure 2:**
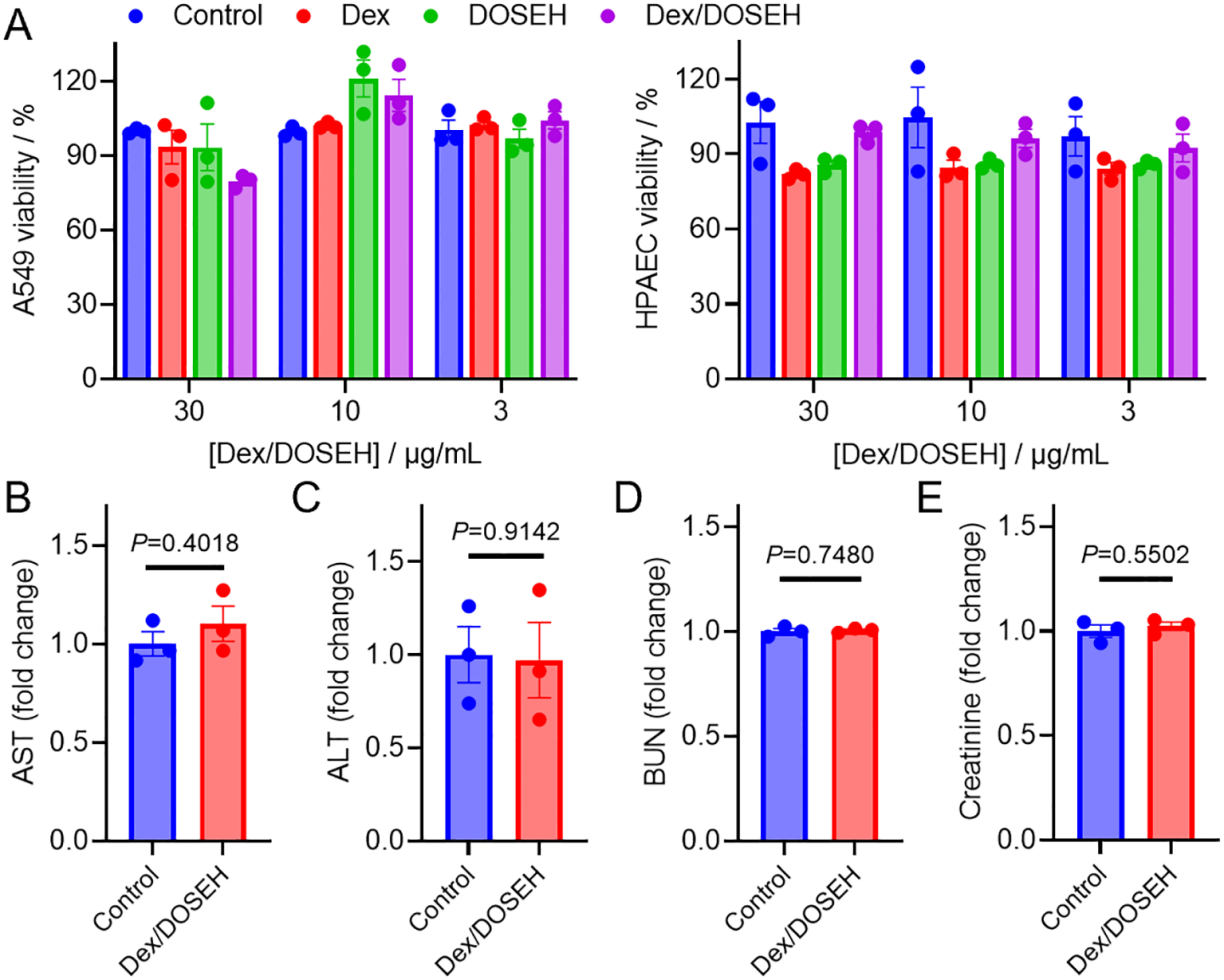
*In vitro* cytotoxicity and *in vivo* biocompatibility of Dex/DOSEH sLNPs. **(A)** Cell viability of A549 and HPAEC cells following incubation with Dex/DOSEH sLNPs. *In vivo* biocompatibility assessment based on plasma biochemical markers, including **(B)** AST, **(C)** ALT, **(D)** BUN, and **(E)** creatinine levels in control and Dex/DOSEH-treated mice. N = 3, mean ± SEM, unpaired two-tailed Student’s t-test.

Our previous studies demonstrated that DOSEH-based sLNPs preferentially transfect pulmonary endothelial and epithelial cells following i.v. administration in mice (28). To evaluate the biocompatibility of the Dex/DOSEH formulation, we first assessed cytotoxicity in A549 human alveolar epithelial cells and primary human pulmonary artery endothelial cells (HPAECs). Across the tested Dex/DOSEH concentration range of 3-30 µg/mL, neither Dex/DOSEH nor the corresponding amounts of free Dex or empty DOSEH sLNPs induced detectable cytotoxicity (**Figure 2A**). Particularly, in A549 cells, Dex/DOSEH treatment resulted in cell viabilities of 104%, 114%, and 80% at 3, 10, and 30 µg/mL, respectively. Similarly, HPAECs maintained high viability following Dex/DOSEH exposure, with viabilities of 92%, 96%, and 99% at 3, 10, and 30 µg/mL, respectively.

To further assess *in vivo* safety, Dex/DOSEH was administered intravenously to mice (0.1 mg Dex/kg body weight), and potential hepatic and renal toxicities were evaluated. Plasma alanine aminotransferase (ALT) and aspartate aminotransferase (AST) levels were measured to assess hepatotoxicity (**Figures 2B, C**), while blood urea nitrogen (BUN) and creatinine levels were used as indicators of renal function (**Figures 2D, E**). At the tested dose, no significant differences were observed between Dex/DOSEH-treated mice and PBS-treated controls for any of these parameters, indicating an absence of detectable acute liver or kidney toxicity. Similarly, no obvious hepatotoxicity or nephrotoxicity was observed in mice treated with free Dex (**Supplementary Figure 1**). Consistent with our previous reports on DOSEH-mediated delivery of mRNA and SsnB, these findings suggest that DOSEH-based sLNPs exhibit a favorable safety profile that is largely independent of the encapsulated cargo, at least within the tested dose range (28, 29). Nevertheless, it is important to note that the toxicity of LNP-based systems can be dose-dependent. The dose used here was selected to match that employed in the endotoxin-induced ALI efficacy studies. A more comprehensive *in vivo* toxicity assessment spanning a broader dose range will be necessary to fully define the safety margins of Dex/DOSEH formulation and to identify any formulation-related toxicities and potential mitigation strategies.

### Dex/DOSEH attenuates proinflammatory cytokine responses

ALI and ARDS are characterized by excessive and dysregulated inflammatory responses at both local and systemic levels (2, 5). A hallmark of disease progression is the robust induction of proinflammatory cytokines. Among these mediators, TNF-α and IL-6 play central roles in the initiation and amplification of inflammation and are closely associated with disease severity and clinical outcomes in ALI/ARDS (38–40). TNF-α promotes activation and recruitment of inflammatory cells, enhances vascular permeability, and amplifies downstream cytokine cascades. IL-6 is a key mediator linking local lung inflammation to systemic inflammatory responses. Elevated IL-6 levels are associated with worse outcomes in ARDS patients, and IL-6 has been shown to promote lung injury through activation of the JAK2/STAT3 signaling pathway, leading to sustained inflammatory signaling and tissue damage (38–40).

**Figure 3:**
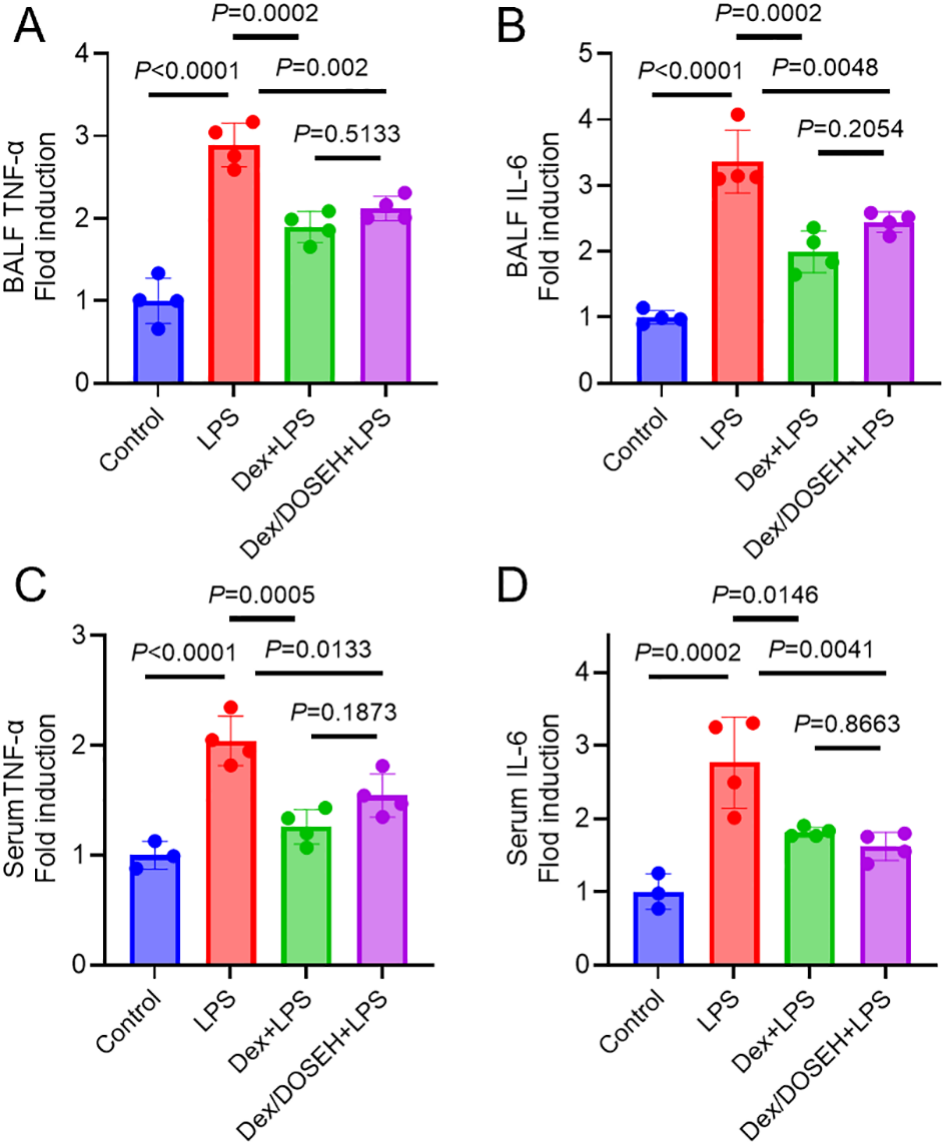
Anti-inflammatory effects of Dex/DOSEH sLNPs in LPS-induced ALI. Levels of **(A)** TNF-α and **(B)** IL-6 in BALF, and serum levels of **(C)** TNF-α and **(D)** IL-6 in control, LPS alone, and LPS with pretreatment of free Dex (0.4 mg Dex/kg body weight) or Dex/DOSEH (0.1 mg Dex/kg body weight). N = 3-4, mean ± SD, one-way ANOVA.

We first evaluated the anti-inflammatory effects of Dex/DOSEH at doses of 0.1, 0.2, and 0.4 mg/kg. A dose of 0.4 mg/kg free Dex was selected based on the DEXA-ARDS and CoDEX trials. Both free Dex and Dex/DOSEH reduced immune cell infiltration and IL-6 levels in bronchoalveolar lavage fluid (BALF) (**Supplementary Figure 2**). Dex/DOSEH at 0.1 mg/kg was selected for subsequent studies, as it produced effects comparable to 0.4 mg/kg free Dex. This finding suggests that lung-targeted delivery via DOSEH sLNPs enhances local drug availability within lung tissue. Such dose sparing represents a potential advantage, as effective suppression of inflammation at lower systemic doses may reduce off-target exposure and drug-associated adverse effects. We then examined TNF-α and IL-6 levels in BALF (**Figures 3A, B**). Compared with control mice, intratracheal LPS challenge resulted in marked elevation of both TNF-α and IL-6, confirming the establishment of a strong inflammatory response. Treatment with free Dex (0.4 mg/kg) significantly reduced the levels of both cytokines. Treatment with Dex/DOSEH (0.1 mg Dex/kg) also led to a comparable reduction in TNF-α and IL-6 expression.

Local pulmonary inflammation in ALI/ARDS can propagate into the systemic circulation, contributing to extrapulmonary organ dysfunction (41, 42). Previous studies have shown that effective suppression of lung inflammation can mitigate systemic inflammatory responses (43–45). Consistent with this concept, we next assessed TNF-α and IL-6 levels in serum (**Figures 3C, D**). As expected, LPS challenge significantly increased circulating levels of both cytokines. Treatment with Dex/DOSEH significantly reduced serum TNF-α and IL-6 concentrations, mirroring the trends observed in BALF. Again, Dex/DOSEH achieved reductions comparable to those observed with free Dex administered at a four-fold higher dose. Collectively, these results demonstrate that Dex/DOSEH effectively suppresses both pulmonary and systemic proinflammatory cytokine responses in an LPS-induced ALI model. The ability to achieve comparable anti-inflammatory efficacy at a reduced drug dose highlights the therapeutic advantages of lung-targeted Dex delivery using DOSEH sLNPs.

### Dex/DOSEH reduces inflammatory cell infiltration and preserves the capillary-alveolar barrier

A defining pathological feature of ALI/ARDS is the recruitment and accumulation of innate immune cells in the lung, particularly neutrophils and macrophages (46, 47). Proinflammatory cytokines are key drivers of leukocyte recruitment by upregulating adhesion molecules, chemokines, and vascular permeability (38). Therefore, effective suppression of cytokine signaling is expected to attenuate immune cell infiltration into the lung and limit inflammation-associated tissue damage (41, 42). To evaluate this effect, we performed cytological analysis of BALF to quantify infiltrating immune cell populations.

**Figure 4:**
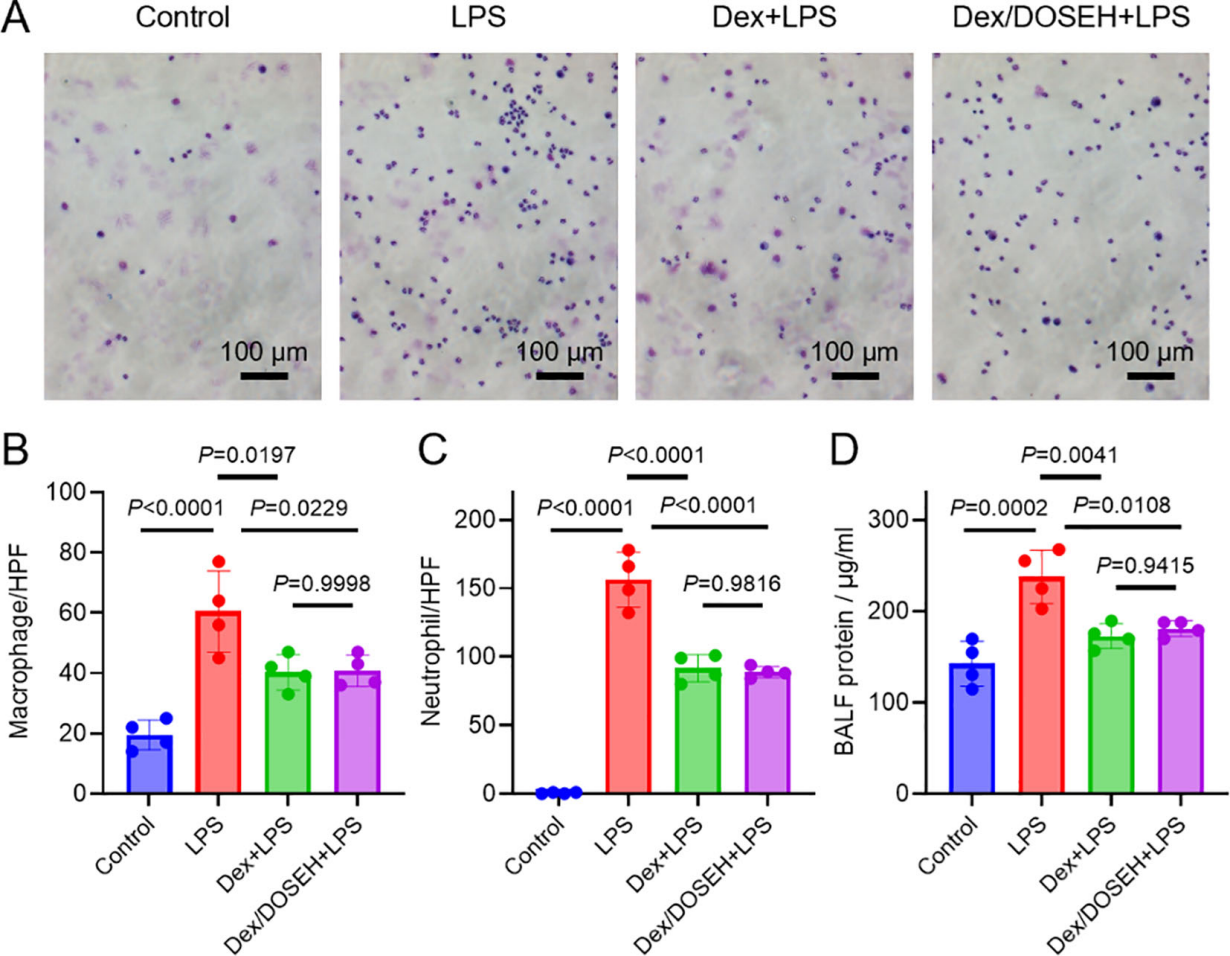
Effects of Dex/DOSEH sLNPs on inflammatory cell infiltration and alveolar permeability. **(A)** Cytological analysis of BALF, with representative images showing cellular populations across different treatment groups, including control, LPS alone, and LPS with pretreatment of free Dex (0.4 mg Dex/kg body weight) or Dex/DOSEH (0.1 mg Dex/kg body weight). Quantification of **(B)** macrophages and **(C)** neutrophils per slide. **(D)** Total protein concentration in BALF measured by the BCA assay. N = 4, mean ± SD, one-way ANOVA.

Following LPS challenge, both macrophage and neutrophil counts in BALF were markedly increased compared with control mice, consistent with robust inflammatory cell recruitment in ALI (**Figures 4A–C**). Treatment with either free Dex (0.4 mg Dex/kg body weight) or Dex/DOSEH (0.1 mg Dex/kg body weight) significantly reduced the accumulation of both inflammatory cell populations. Notably, Dex/DOSEH achieved comparable efficacy at a substantially lower dose, indicating that lung-targeted delivery enhances the inhibitory effect of Dex on inflammatory cell recruitment in this model.

In addition to immune cell infiltration, disruption of the capillary-alveolar barrier is a central event in ALI/ARDS pathogenesis (2, 6). Barrier dysfunction results from inflammatory signaling-mediated injury to endothelial and epithelial layers, leading to increased vascular permeability, extravasation of plasma proteins into the alveolar space, and pulmonary edema. To assess barrier integrity, total protein concentration in BALF was measured. Consistent with barrier disruption, LPS exposure led to a significant increase in BALF protein concentration compared with controls (**Figure 4D**). Both free Dex and Dex/DOSEH treatments significantly reduced BALF protein levels, indicating attenuation of vascular leakage and partial preservation of capillary-alveolar barrier function. These findings suggest that suppression of inflammation by Dex/DOSEH not only limits immune cell infiltration but also mitigates downstream barrier dysfunction. Taken together, these results demonstrate that Dex/DOSEH effectively reduces macrophage and neutrophil infiltration into the lung and preserves capillary-alveolar barrier integrity in LPS-induced lung inflammation. These effects likely reflect coordinated suppression of inflammatory signaling and vascular permeability.

### Dex/DOSEH attenuates lung tissue injury

**Figure 5:**
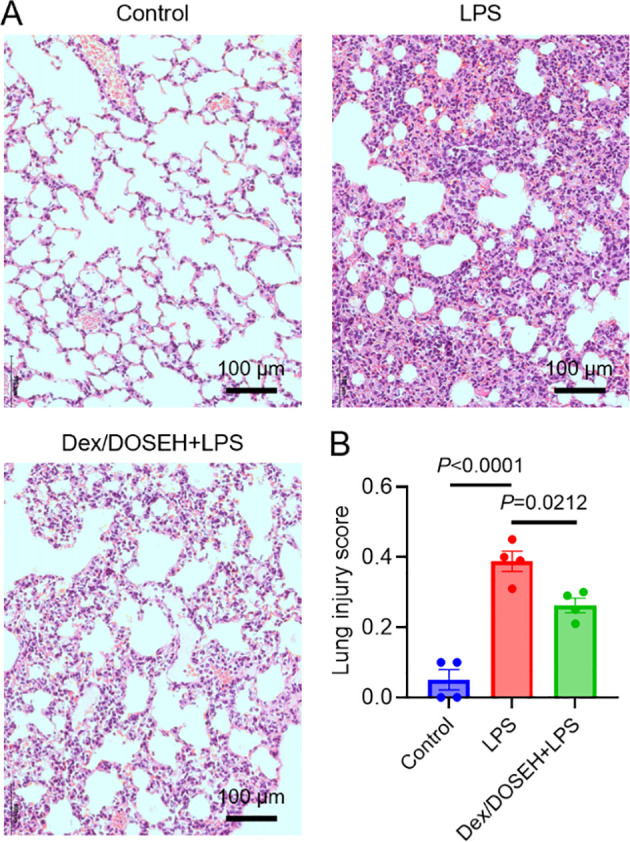
Dex/DOSEH treatment alleviates lung tissue injury in mice. **(A)** H&E staining was performed to assess lung injury across different treatment groups, including control, LPS alone, and LPS with pretreatment of Dex/DOSEH (0.1 mg Dex/kg body weight). **(B)** A semi-quantitative lung injury score was determined. N = 4, mean ± SEM, one-way ANOVA.

In ALI/ARDS, excessive production of proinflammatory cytokines, sustained recruitment of immune cells, and disruption of the capillary-alveolar barrier act in a coordinated manner to drive lung tissue injury. To evaluate the impact of Dex/DOSEH on lung tissue injury, histological examination of lung sections was performed using hematoxylin and eosin (H&E) staining (**Figure 5A**). Compared with control lungs, LPS-challenged mice exhibited pronounced pathological changes, including extensive neutrophil infiltration in both alveolar and interstitial spaces, hyaline membrane formation, accumulation of proteinaceous debris within the airspaces, and marked thickening of alveolar septa. These findings are consistent with severe inflammatory injury and barrier disruption. In contrast, lungs from Dex/DOSEH-treated mice showed a clear attenuation of these pathological features. Neutrophil accumulation was reduced, alveolar structure was better preserved, and the extent of hyaline membrane formation, airspace debris, and septal thickening was markedly reduced. Semi-quantitative histological scoring further supported these observations, with LPS-challenged mice exhibiting an average lung injury score of approximately 0.39, whereas Dex/DOSEH treatment reduced the score to approximately 0.26 (**Supplementary Table 1**, **Figure 5B**). Collectively, the observed reduction in tissue injury is consistent with the coordinated suppression of proinflammatory cytokine production, immune cell infiltration, and vascular leakage.

## Summary

In summary, we developed a ligand-free, lung-targeting sLNP formulation of Dex (Dex/DOSEH) for i.v. administration. Dex/DOSEH exhibited favorable physicochemical properties and selectively accumulated in lung tissue following systemic administration. The formulation showed good biocompatibility both *in vitro* and *in vivo* assessments. In an LPS-induced ALI mouse model, treatment with Dex/DOSEH significantly reduced the expression of key proinflammatory cytokines (TNF-α and IL-6) in both BALF and serum. In addition, Dex/DOSEH limited infiltration of macrophages and neutrophils into the lung and preserved capillary-alveolar barrier integrity. Consistent with these findings, lung histological analysis revealed that Dex/DOSEH attenuated lung tissue injury and improved overall lung architecture. Collectively, these results demonstrate that Dex/DOSEH enables effective lung-targeted delivery of Dex and achieves robust anti-inflammatory efficacy, highlighting its potential as a therapeutic strategy for ALI/ARDS. Nevertheless, several limitations warrant further investigation. Future studies will focus on optimizing sLNP formulation parameters to improve drug loading efficiency and lung delivery specificity (48); conducting dose-dependent and long-term toxicity evaluations to fully define safety margins (49); and assessing the therapeutic efficacy of Dex/DOSEH across different stages and etiologies of ALI/ARDS (6). In addition, the present findings are derived from murine studies, and given the physiological and immunological differences between mice and humans, further validation in animal models more closely related to humans is required prior to clinical translation (50).

## Data Availability

The original contributions presented in the study are included in the article/**Supplementary Material**. Further inquiries can be directed to the corresponding authors.
