# Supplementary material for: Lung-specific sulfonium lipid nanoparticle formulation of dexamethasone suppresses endotoxin-induced lung inflammation
Source: Front Immunol. 2026 May 29;17:1821659. doi: 10.3389/fimmu.2026.1821659 (PMC13259906; doi:10.3389/fimmu.2026.1821659)
Supplement: Supplementary file 1 [file DataSheet1.docx]

**Supporting Information**

**Lung-Specific Sulfonium Lipid Nanoparticle Formulation of Dexamethasone Suppresses Endotoxin-Induced Lung Inflammation**

Yuqin Men^1^, Chunyan Wang^2^, David O. Popoola^1^, Zhi Cao^1^, Weikun Tian^2^, Robert N. Cooney^2,3^, Qinghe Meng^2,3*^, and Yamin Li^1,3*^

^1^Department of Pharmacology, State University of New York, Upstate Medical University, Syracuse, NY 13210, USA

^2^Department of Surgery, State University of New York, Upstate Medical University, Syracuse, NY 13210, USA

^3^Sepsis Interdisciplinary Research Center (SIRC), State University of New York, Upstate Medical University, Syracuse, NY 13210, USA

*Corresponding Author: Q. Meng (mengq@upstate.edu), Y. Li (liyam@upstate.edu)

**Table S1.** Lung injury scoring evaluation.

| Parameter | Score per field | | |
| --- | --- | --- | --- |
|  | 0 | 1 | 2 |
| A. Neutrophils in the alveolar space | none | 1-5 | >5 |
| B. Neutrophils in the interstitial space | none | 1-5 | >5 |
| C. Hyaline membranes | none | 1 | >1 |
| D. Proteinaceous debris filling the airspaces | none | 1 | >1 |
| E. Alveolar septal thickening | <2x | 2x-4x | >4x |


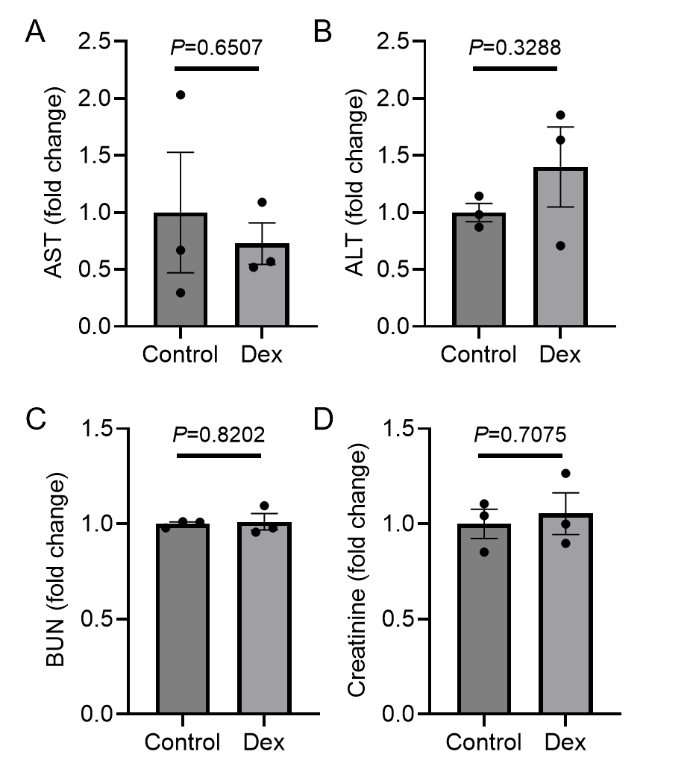


**Figure S1.** In vivo biocompatibility of free Dex (0.4 mg/kg). In vivo biocompatibility assessment based on blood biochemical markers, including (A) AST, (B) ALT, (C) BUN, and (D) creatinine levels in control and free Dex-treated mice (0.4 mg/kg). N = 3, mean ± SEM, unpaired two-tailed Student’s t-test.


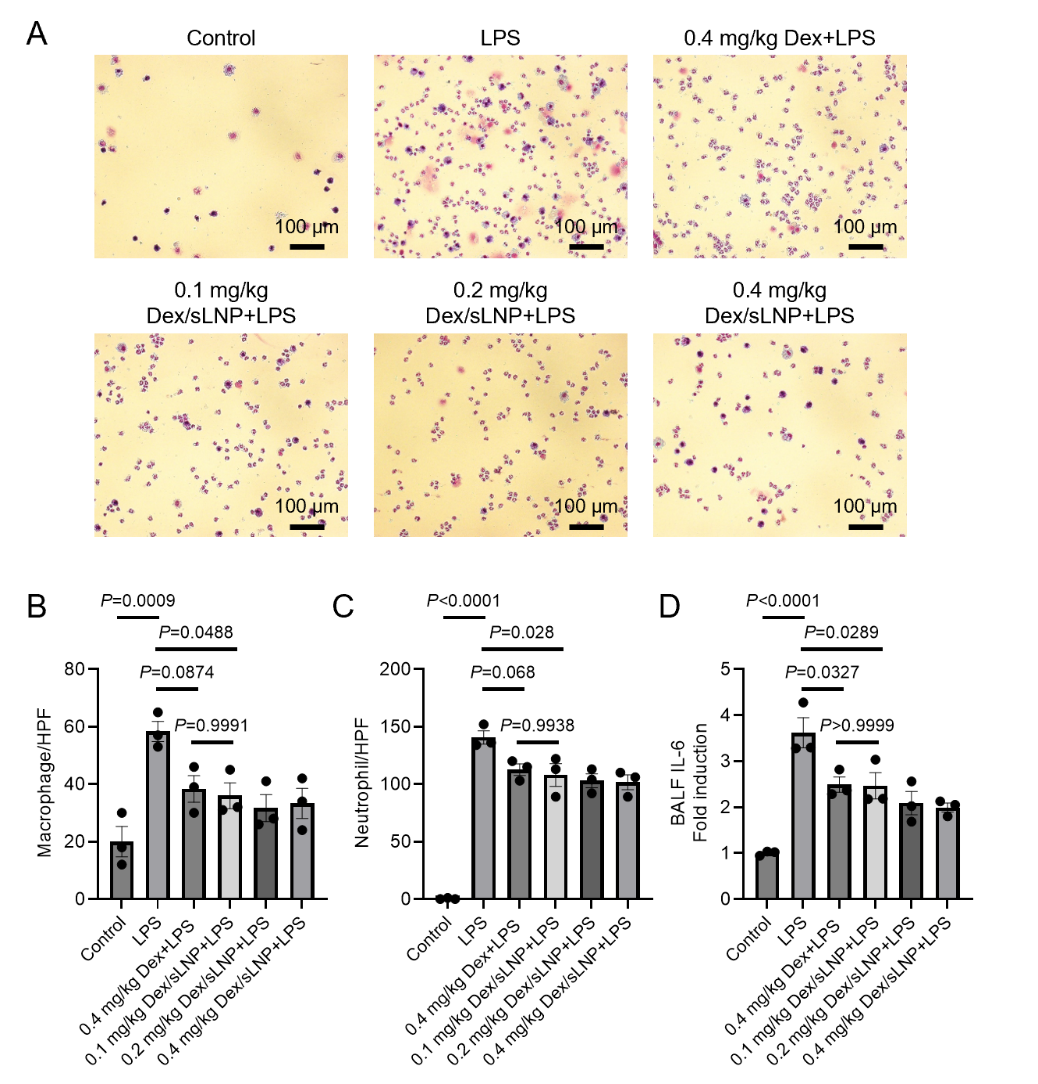


**Figure S2.** Effects of free Dex and Dex/DOSEH sLNP formulation on inflammatory cell infiltration and cytokine expression. (A) Cytological analysis of BALF, with representative images showing cellular populations across different treatment groups, including control, LPS alone, and LPS with pretreatment of free Dex (0.4 mg Dex/kg body weight) or Dex/DOSEH (0.1, 0.2, and 0.4 mg Dex/kg body weight). Quantification of (B) macrophages, (C) neutrophils per slide, and (D) IL-6 fold induction in BALF. N = 3, mean ± SEM, one-way ANOVA.
